# Psychometric properties and validation of the revised Chinese Medication Literacy Scale for Hypertensive Patients (C-MLSHP-R)

**DOI:** 10.3389/fcvm.2022.976691

**Published:** 2022-09-06

**Authors:** Ning Qin, Yinglong Duan, Ziqiang Yao, Shuangjiao Shi, Haoqi Liu, Xiao Li, Feng Zheng, Zhuqing Zhong

**Affiliations:** ^1^Nursing Department, The Third Xiangya Hospital, Central South University, Changsha, China; ^2^Xiangya School of Nursing, Central South University, Changsha, China; ^3^Emergency Department, The Third Xiangya Hospital, Central South University, Changsha, China; ^4^Cardiology Department, The Third Xiangya Hospital, Central South University, Changsha, China; ^5^Key Laboratory of Medical Information Research, College of Hunan Province, Central South University, Changsha, China

**Keywords:** hypertension, medication literacy, assess, validity, reliability

## Abstract

**Background:**

Medication literacy is one of the key indicators that can affect the self-management of medications and medication safety. This study aimed to revise the Chinese Medication Literacy Scale for hypertensive patients (C-MLSHP) and test the reliability and validity of the revised scale.

**Methods:**

We revised the C-MLSHP by several methods, i.e., focus group discussion, expert consultation, patient interview, and pilot study, based on the established evaluation index system of medication literacy for hypertensive patients. Then, a formal survey using the revised Chinese Medication Literacy Scale for hypertensive patients (C-MLSHP-R) was carried out on hypertensive patients from hospitals and community healthcare centers in Changsha city of China to test its reliability and validity. The reliability was evaluated with Cronbach's α coefficient, split-half reliability, and test–retest reliability. The validity was evaluated with content validity, construct validity, convergent validity, discriminant validity, and criterion-related validity.

**Results:**

The C-MLSHP-R contained 18 items within four domains, i.e., the knowledge domain included four items, the attitude domain had three items, the skill domain involved seven items, and the practice domain included four items. A total of 339 hypertensive patients participated in the formal survey. The results showed that the Cronbach's α coefficient of C-MLSHP-R was 0.802, and for each domain ranged from 0.639 to 0.815. The split-half reliability coefficient of C-MLSHP-R was 0.709, and for each domain ranged from 0.648 to 0.792. The test–retest reliability coefficient of C-MLSHP-R was 0.851, and for each domain ranged from 0.655 to 0.857. The I-CVI of each item ranged from 0.833 to 1.000, the S-CVI/Ave of C-MLSHP-R was 0.981, the S-CVI/UA was 0.889, and for each domain ranged from 0.958 to 1.000. Confirmatory factor analysis results showed that the model fitted well. The convergent validity of C-MLSHP-R was acceptable, and the discriminant validity was good. The criterion coefficient between C-MLSHP-R and C-MLSHP was 0.797, and for each domain ranged from 0.609 to 0.755.

**Conclusion:**

Compared with C-MLSHP, the C-MLSHP-R with 18 items was much shorter for measuring, and had decreased reliability within the acceptable range and better validity, which was more appropriate and time-saving to assess the medication literacy level for hypertensive patients scientifically and conveniently.

## Introduction

Hypertension is the predominant modifiable risk domain for cardiovascular disease (1), leading to a heavy economic burden on countries and families (2, 3). However, hypertension management issues are perceived as “serious” and may last for a long time. Until now, the situation of hypertension management was not optimistic across the world, given the low rate of hypertension awareness, treatment, and control, especially in low- and middle-income countries (4). In China, the hypertension awareness, treatment, and control rates among adults in 2012–2015 were 46.9, 40.7, and 15.3% (5), while among people of age 35–75 years in the community in 2014–2017 were 36.0, 22.9, and 5.7%, respectively (6). Medication therapy was one of the most vital and effective management measures to control blood pressure and retard the disease progression. Medication literacy can reveal the patient's medication knowledge, attitude, skill, and practices comprehensively. It is the necessary literacy for hypertensive patients to manage self-medication. Identifying medication literacy levels of hypertensive patients is essential for health care workers and managers to formulate comprehensive medication strategies to optimize blood pressure control rate (7). Previous studies showed that adequate knowledge about hypertension was one important reason for hypertensive patients to correct poor lifestyles and develop heart-healthy lifestyles (8, 9). Additionally, the important barriers to the low treatment and control rates for hypertension were as follows: lack of knowledge and skill about taking antihypertensives, as well as medication necessity belief, poor adherence to antihypertensives, and irregular medication-taking behavior (10). Meanwhile, medication literacy was identified as one independent predictor of medication adherence (11), and those with high medication adherence were about five times more likely to have controlled blood pressure compared to those with low medication adherence (12). Therefore, medication literacy has a very vital impact on hypertension management.

Medication literacy was first coined in the medication safety report of the UK government in 2005 and was formally proposed by Raynor in 2008 (13). Royal pointed out that addressing medication literacy was a pharmacy practice priority, and explained it as “a person's accessibility and understandability of spoken and written information to make decisions about the correct medication for oneself, allowing the safe and effective medication use” (14). Sauceda and his colleagues defined medication literacy as “the ability of individuals to access, understand, and practice basic medication information safely and appropriately” based on the definition of health literacy in 2012 (15). In 2018, Pouliot et al. (16) proposed the working definition of medication literacy based on four-round Delphi expert consultation. It was “the degree to which individuals can obtain, comprehend, communicate, calculate, and process patient-specific information about their medications to make informed medication and health decisions aiming for safe and effective medication use, regardless of the mode by which the content is delivered (e.g., written, oral, and visual).” Gentizon et al. (17) indicated that medication literacy could be varying in different circumstances and cases, so its concept and connotation should not be uniform for different populations and medical cultures. It would be critical to make necessary adjustments according to specific situations for optimizing the accuracy of measurement and applicability in adapted measures.

Several self-reported tools have been developed to assess the levels of medication literacy so far. Among them, tools applicable to the whole population mainly included Medication Literacy Questionnaire (18), Medication Literacy Assessment in Spanish and English (MedLitRxSE) (15), Chinese Medication Literacy Measurement (ChMLM) (19, 20), and Functional Medication Literacy Tool (21). In addition, there are several tools specific to the targeted population that are used for assessing the levels of medication literacy [e.g., pregnant women (22) and hemodialysis patients (23)] and specific drugs [e.g., non-steroidal anti-inflammatory drugs (24) and cold drugs (25)]. The definitions of medication literacy in the development process of different tools varied widely, most of whose content mainly focus on the ability of individuals to read, understand, and process medical information, especially medication labels and doctors' prescriptions. Some tools are even developed without a definition of medication literacy (17). Thus, the measurement content and scope for medication literacy varies a lot with different tools. To our knowledge, the aforementioned tools are not applicable for measuring the specific medication knowledge, attitude, skill, and practice of hypertensive patients. Therefore, a large gap would occur between the assessment results and the actual values if a hypertension-specific scale for medication literacy were not developed.

Zhong et al. (26) developed the theoretical framework of medication literacy with four operationalized elements (knowledge, attitude, skill, and practice). Furthermore, the concept of medication literacy specific for hypertensive patients was identified based on the following theories of health literacy, planned behavior, knowledge-attitude-practice, and the concept of Chinese resident health literacy. It was finally defined as “the ability of hypertensive patients to obtain, understand, and evaluate hypertensive disease and drug information to make appropriate medication decisions and take medication-related actions.” Meanwhile, the evaluation index system of medication literacy for hypertensive patients was established based on two-round Delphi expert consultation, on the basis of which the Chinese Medication Literacy Scale for Hypertensive Patients (C-MLSHP) was developed and validated (27). The C-MLSHP with 37 items involving four domains (knowledge, attitude, skills, and practice) and 11 sub-domains was a relatively satisfactory tool to assess medication literacy for hypertensive patients (28, 29). The Cronbach's α coefficient of the scale was 0.849, the split-half reliability coefficient was 0.893, and the test–retest correlation coefficient was 0.968.

However, the explained variation of the scale in exploratory factor analysis (EFA) was 51.42% (<60%), and several fit indices for the four-domain model were not satisfied (GFI = 0.804 <0.90, AGFI = 0.777 <0.90, IFI = 0.746 <0.90), indicating a moderate construct validity of C-MLSHP. Meanwhile, a total of 37 items was time-consuming for patients to complete, especially for the elderly, making it poorly generalizable and inferiorly applicable in real-world studies. Thus, this study aimed to revise and reduce the items for the C-MLSHP and test the reliability and validity of the revised version. It was an indispensable step to provide a more effective, pragmatic, convenient, and useful tool for healthcare workers and managers to screen out hypertensive patients with under desirable medication literacy levels.

## Methods

The revised Chinese Medication Literacy Scale for hypertensive patients (C-MLSHP-R) was based on the five-stage approach proposed by McDowell and Jenkinson: scale development, scale modifications, clarity verification, item simplification, and scale validation (30). This study was approved by the Institutional Review Board of the Third Xiangya Hospital, CSU (I 21072).

### C-MLSHP-R development

A focus group consisted of all the members that participated in the development process of the C-MLSHP (one chief nurse, one deputy chief nurse, one chief pharmacist, two nurses in charge, one senior nurse, and three postgraduate students). All group members reviewed the previous studies regarding the performance of the C-MLSHP based on the evaluation index system of medication literacy in hypertensive patients, as well as helpful feedback from patients and suggestions for improvement (11, 26–29). After a three-round focus group discussion, the comments were uniform finally, and the preliminary revision scale with 26 items consisting of the knowledge domain (four multiple-choice items), attitude domain (seven items), skill domain (seven items), and practice domain (eight items) was developed. Compared to the 37-item C-MLSHP, the specific revisions of the preliminary revision scale were stated as follows.

For the knowledge domain, we revised with four multiple-choice items instead of the prior nine items. Three previous items belonging to one of the sub-domains of knowledge assessing patients' knowledge about hypertension disease were merged in accordance with the added content evaluating patients' knowledge about blood pressure control targets, such as comorbid patients and the elderly. Another two items assessing patients' knowledge about antihypertensive drugs were merged into one multiple-choice item. The remaining four items pertaining to one of the sub-domains of knowledge assessing patients' knowledge about hypertension treatment were merged into one multiple-choice item.

For the attitude domain, seven items were replaced with the previous eight items. We revised items that were not related to medication to ensure the correlation between each item with its belonging domain. Two items assessing patients' attitudes toward taking antihypertensive drugs were merged into one item. Provided that most of the commonly used antihypertensive drugs were long-acting drugs and the clinical effect of occasionally missing the drugs was unclear, the item with regard to patients' attitude to missed drugs was replaced with an item related to their attitude to long-term antihypertensive treatment.

For the skill domain, seven items were the same as that in the original scale. We updated the drug prescription in the offered cases combining it with actual clinical drug prescriptions. The aspirin enteric coated tablet used in the original scale was deleted as it was a non-antihypertensive drug. We revised the drug instruction with one for commonly used antihypertensive, i.e., amlodipine besylate tablet. Meanwhile, the item measuring patients' comprehension of antihypertensive prescriptions was replaced with an item testing their ability to recognize adverse drug reactions.

For the practice domain, eight items were replaced with the original 13 items. Three items assessing the frequency, precautions, and records of self-measured blood pressure monitoring were revised with one item. Another three items assessing the drug information-seeking behavior (frequency and time) and patients' advocacy behavior for the used antihypertensives were replaced with one item. The remaining four items assessing patients' adherence to taking antihypertensives were revised with two items. Finally, we added one item measuring patients' adherence to regular outpatient visits.

### C-MLSHP-R modifications

Six experts specialized in clinical medicine, nursing care, pharmacology, or public health education were invited to participate in a two-round expert consultation. Consultation letters including three parts of introduction, consultation form on scale items, and basic information of experts were delivered to experts by e-mail or face-to-face. In the part of the consultation form, we invited experts to make comments on each item and evaluate the correlation between each item and its belonging sub-domain with a Likert 4-grade score of 1–4 (from “not relevant” to “very relevant”). Of the six experts, two were certified as clinical medical experts, two were nursing specialists, one was a pharmacy specialist, and one was a public health specialist. Among them, two were men, four were women, and the average age was (45.50 ± 8.02) years. Five experts had over 10 years of work experience in their field, and one expert had over 5 years of experience. We revised each item by combining expert comments with the Item-Content Validity Index (I-CVI). To adjust the selection bias of scoring among experts, the random consistency was examined using the Kappa value (K^*^). The evaluation criteria were as follows: poor (K^*^ <0.40), moderate (K^*^: 0.40–0.59), good (K^*^: 0.60–0.74), and excellent (K^*^ > 0.74) (31). In the first round of expert consultation, item A4, item A5, item P4, and item P8 were considered to show poor correlation with their corresponding domains (I-CVI <0.78). The random consistency for the above four items was also reported as poor or moderate. So, we deleted them. Meanwhile, we revised item K2 and item K3, as well as one response option for item K4 according to experts' suggestions. In the second round, the I-CVI (1.000) and K^*^(1.000) values for all the items except for item K2 and item P1 were excellent. The I-CVI (0.833) and K^*^(0.816) values for item K2 and item P1 were good. Thus, the remaining 22 items were retained for the primary C-MLSHP-R. There were four domains in this scale as follows. The knowledge domain had four multiple-choice items, the attitude domain had five items, the skill domain had seven items, and the practice domain had six items.

### Clarity verification

A total of 15 hypertensive patients were selected in the interview by using a quota sampling method. The interview aimed to evaluate the readability and understandability of each item with a Visual Analog Scale (VAS) from 0 (unclear) to 10 (very clear) (32). When an item received <7 points, patients would be further invited to provide specific suggestions for item modifications. The interviews were performed in a quiet environment within 30 min for hypertensive patients with no relevant treatment. Of the 15 patients, eight were women and seven were men. The average age was (74.93 ± 10.06) years. Four patients had an education level of primary school or below, five of junior middle school, four of senior high school or technical secondary school, one of junior college, and one of college or above. The average duration of having hypertension for all patients was (11.75 ± 9.64) years, and the average duration of taking antihypertensives was (17.82 ± 11.49) years. The results showed that the primary C-MLSHP-R had good clarity with all items ≥7 points and was appropriate to apply in the pilot study.

### Item simplification

The pilot study was conducted from June to August 2021, and statistical analysis was performed to test and simplify the items. We selected hypertensive patients in wards and clinics of a tertiary hospital and two community health service centers in Changsha city of China with a convenient sampling method. The inclusion criteria were as follows: (a) individuals over 18 years of age; (b) patients were diagnosed with hypertension according to 2018 Chinese Guidelines for the Management of Hypertension (33); (c) currently taking or had taken antihypertensives in the past 3 months; (d) normally functioning in communicating and reading; and (e) agreeing to participate in this study. The exclusion criteria were as follows: (a) patients with mental disorders, hypertension crisis, or hypertensive encephalopathy; and (b) currently participating in or have participated in hypertension-related intervention programs in the past 30 days. The questionnaire included two parts of general information (gender, age, education, marital status, occupation, etc.) and the primary C-MLSHP-R with 22 items. Generally, the sample size for the pilot study should be 5–10 times the number of items on the used scale (34), so the minimum sample size calculated in our pilot study was 110 samples. Data collection was conducted face-to-face by three experienced and trained investigators. For item simplification, Cronbach's α reliability analysis, item discrimination analysis, and correlation coefficient method were used, and the results were described below.

A total of 115 hypertensive patients participated in the pilot investigation, and 110 valid questionnaires were received with an effective response rate of 95.65%. The results showed that the Cronbach's α coefficient of the total scale was 0.791, and the Cronbach's α coefficient increased when deleting item A1 (0.794), item A2 (0.804), and item P5 (0.802), so these items were considered to be deleted. The results of item discrimination analysis showed no significant difference between the high and low score groups in item A1, item A2, and item P5 (*P* > 0.05), indicating poor discrimination of these items. Thus, item A1, item A2, and item P5 were ready to be deleted. The results of the correlation coefficient method showed that the correlation coefficients between items K1–K4 and the knowledge domain ranged from 0.707 to 0.851 (*P* < 0.001), while the correlation coefficient between item K3 and the skill domain was more than 0.50. The correlation coefficients between item A1–A2 and the attitude domain were <0.50, which were considered to be deleted. The correlation coefficients between items S1.1–S2.4 and the skill domain ranged from 0.594 to 0.759 (*P* < 0.001), and the correlation coefficients between items S1.1–S2.4 and other domains were all <0.50. The correlation coefficients between item P2, item P5, and the practice domain were <0.50. Thus, item K3, item A1, item A2, item P2, and item P5 were considered to be deleted. In summary, item A1, item A2, item P2, and item P5 were deleted by combining the above three methods. Finally, there were four multiple-choice items on knowledge, three items on attitude, seven items on skill, and four items on practice in the C-MLSHP-R.

### C-MLSHP-R validation

The formal survey was conducted from September to November 2021. We selected hypertensive patients in wards and clinics of two tertiary hospitals and two community health service centers in Changsha city of China with a convenient sampling method. The inclusion and exclusion criteria were the same as that in the pilot study. The sample size in confirmatory factor analysis (CFA) should be larger than 200 and greater than the sample size in EFA (>100) (35), so the minimum sample size calculated in the formal investigation was 334 considering 10% invalid questionnaires. Data collection was conducted face-to-face by three experienced and trained investigators.

### Instruments

#### Socio-demographic information

Socio-demographic information questionnaire included gender, age, education, residence type, marital status, occupational situation, family history of hypertension, duration of hypertension, and duration of taking antihypertensives.

#### C-MLSHP-R

The C-MLSHP-R had 18 items with four domains (See Supplementary material 1). The knowledge domain included four multiple-choice items from K1 to K4, and each item was responded to with a 5-point Likert scale scored zero to four. “I don't know” scoring zero and the other four options describing patients' knowledge about hypertensive-with-medication scored one, respectively. The attitude domain included three items from A1 to A3, and the practice domain had four items from P1 to P4 and responded with a 0–4 Likert scale. Items A1–A3 scored reversely. The skill domain included seven items from item S1.1 to item S2.4. For responding to them, choosing the correct answer was assigned 1 point, and an incorrect answer or “I do not know” was given 0 points. A sum of the C-MLSHP-R scores ranged from 0 to 51. Higher scores meant a higher level of medication literacy for hypertensive patients. The comparison of the medication literacy evaluation index system and scale items between C-MLSHP-R and C-MLSHP was shown in Supplementary material 2.

### Data analysis

#### Descriptive statistics

Mean values and standard deviations (SDs) were used for consecutive variables, while frequencies and percentages (%) were used for categorical variables describing patients' characteristics.

#### Reliability test

Internal consistency and temporal stability were tested for reliability. The former consisted of Cronbach's α coefficient and split-half reliability coefficient, and the latter was evaluated by test–retest reliability. When the Cronbach's α of the total scale was ≥0.80, the Cronbach's α of each domain was ≥0.70, and the split-half reliability coefficient of the total scale was ≥0.70, thus acceptable internal consistency reliability for the scale was reached (36, 37). We selected 30 hypertensive patients to fill in the scale twice at a 2-week interval, and the Pearson's correlation coefficient of the total scale between measures was calculated. Pearson's correlation coefficient ≥0.60 showed acceptable temporal stability (38).

#### Validity test

Content validity, construct validity, convergent validity, discriminant validity, and criterion-related validity were tested for the validity of this scale. Content validity aims to test the degree to which the items measured matched with what was expected to be measured by a scale. The content validity index of the scale was calculated according to the results of the second round of expert consultation. When I-CVI ≥ 0.78, S-CVI/UA ≥ 0.80, and S-CVI/Ave ≥ 0.90, the content validity was good (31). Construct validity was tested using EFA and CFA. For EFA, the Kaiser-Meyer-Olkin (KMO) value and Bartlett's test of sphericity were used to assess the applicability of factor analysis. The KMO value ≥0.60 and Bartlett's test of sphericity significantly (*P* < 0.05) indicated the suitability of factor analysis (39). Then, principal component analysis and the varimax rotation method were used for extracting common factors (eigenvalues >1) for the scale. Factor loadings for items >0.40 and cumulative variance contribution rate >60% indicated good construct validity. CFA was applied to determine model fit with AMOS 25.0. Model fit indices evaluated in this study were as follows: chi-square value degrees of freedom ratio (χ^2^/df), Root Mean Square Error of Approximation (RMSEA), Goodness of Fit Index (GFI), Adjusted Goodness of Fit Index (AGFI), Comparative Fit Index (CFI), Incremental Fit Index (IFI), Tucker-Lewis Index (TLI), Parsimonious Comparative Fit Index (PCFI), and Parsimonious Normed Fit Index (PNFI). The criteria for a good fit were GFI, AGFI CFI, IFI, and TLI values above 0.90; PCFI and PNFI values above 0.50; and RMSEA values <0.50 (40). Convergent validity reflects the degree to which each item belongs to its corresponding domain in the actual measurement. Convergent validity was assessed by the average variance extracted (AVE), standardized factor loading (SFL), and composition reliability (CR). When SFL > 0.50, CR > 0.70, and AVE > 0.50, the convergent validity was good (40). Discriminant validity reflects the extent to which each item belongs to the remaining domains of the scale in actual measurement. The square root of AVE greater than the correlations among domains indicated good discrimination validity (41). Criterion-related validity reflects the extent to which the score of the adapted scale correlated with the previously common-used measure. We invited hypertensive patients to complete the C-MLSHP-R and the C-MLSHP at the same time. Taking the C-MLSHP as the validity criterion, the Pearson correlation coefficient between the C-MLSHP-R and the C-MLSHP was calculated. A validity coefficient larger than 0.60 indicated good criterion-related validity (42).

## Results

### Sample characteristics

The questionnaires were sent out to 343 hypertensive patients, and a total of 339 valid questionnaires were received with an effective response rate of 98.83%. Among the 339 hypertensive patients, 221 (65.19%) were men and 118 (34.81%) were women. The average age was (63.83 ± 11.80) years. The majority received education in junior middle school (33.04%) or senior high school or technical secondary school education (26.84%). A total of 197 patients (58.11%) were from urban areas, while 142 patients (41.89%) came from rural areas. Most (93.22%) of them were single. More than half (50.1%) were retired, and some (18.29%) were employed or took part-time jobs. Nearly, three-fifths (58.70%) had a family history of hypertension. The average duration of hypertension for all participants was (11.75 ± 9.64) years, and the majority had a duration of 5–10 years (25.37%) or more than 10 years (42.18%). The average duration of taking antihypertensives was (10.27 ± 9.10) years, with the majority having taken antihypertensives for 5–10 years (24.78%) or more than 10 years (35.10%) (Table 1).

**Table 1:** Sample characteristics (*N* = 339).

**Variables**	**Group**	***n***	**%**
Gender	Male	221	65.19
	Female	118	34.81
Age (years)	18–45	21	6.19
	46–60	101	29.79
	60–75	165	48.67
	>75	52	15.34
Education	Primary school or below	81	23.89
	Junior middle school	112	33.04
	Senior high school or secondary technical school	91	26.84
	Junior college	27	7.96
	Undergraduate and above	28	8.26
Residence type	Urban	197	58.11
	Rural	142	41.89
Marital status	Single	23	6.78
	No single	316	93.22
Occupational situation	Full-time job or part-time job	62	18.29
	Unemployed	107	31.56
	Retired	170	50.15
Family history of hypertension	Yes	140	41.30
	No	199	58.70
Duration of hypertension (years)	<1	38	11.21
	1–3	33	9.73
	3–5	39	11.50
	5–10	86	25.37
	>10	143	42.18
Duration of taking antihypertensives (years)	<1	45	13.27
	1–3	44	12.98
	3–5	47	13.86
	5–10	84	24.78
	>10	119	35.10

### Reliability of C-MLSHP-R

The Cronbach's α coefficient of the total scale was 0.802 (>0.80), and the Cronbach's α coefficient of each domain ranged from 0.639 to 0.815 (>0.60), indicating acceptable internal consistency of C-MLSHP-R.

The split-half reliability coefficient of the total scale was 0.709 (>0.70), and the split-half reliability coefficient of each domain ranged from 0.648 to 0.792 (>0.60), indicating acceptable internal consistency of C-MLSHP-R.

The test–retest reliability of the total scale was 0.851 (>0.80), and the test–retest reliability of each domain ranged from 0.655 to 0.857 (>0.60), indicating good stability of C-MLSHP-R. Details are shown in Table 2.

**Table 2:** The reliability coefficients of the total scale and among domains of C-MLSHP-R (*N* =339).

**Domains**	**Cronbach's α**	**Split-half reliability**	**Test-retest reliability**
Knowledge	0.679	0.714	0.857**
Attitude	0.788	0.792	0.764**
Skill	0.815	0.752	0.822**
Practice	0.639	0.648	0.655**
Medication literacy	0.802	0.709	0.851**

^**^P < 0.001.

### Validity of C-MLSHP-R

#### Content validity

According to the results of the second round of expert consultation, the I-CVI of each item ranged from 0.833 to 1.000 (>0.78), the S-CVI/UA of the total scale was 0.889 (>0.80), the S-CVI/UA for the four domains were 0.958, 1.000, 1.000, and 0.958 (>0.80), respectively, and the S-CVI/Ave of the total scale was 0.981(>0.90), indicating good content validity of C-MLSHP-R.

#### Construct validity

We divided the collected data into two parts by randomly extracting 110 samples from the total samples using SPSS. They were applied for EFA, and the remaining 229 samples were analyzed for CFA.

#### Exploratory factor analysis

The KMO value and Bartlett's test of sphericity showed satisfactory results (KMO = 0.843; χ^2^ = 728.399, *P* < 0.001). EFA extracted four common domains (eigenvalues >1), and the cumulative variance contribution rate was 60.002%. The total explained variances for each domain ranged from 11.297 to 21.369%. After varimax rotation, the factor loadings of all items ranged from 0.413 to 0.888 (> 0.40) except for item A4 (0.354), indicating good construct validity (Table 3).

**Table 3:** The results of EFA for C-MLSHP-R (*n* = 110).

**Items**	**Domains**			
	**1**	**2**	**3**	**4**
K1	0.723			
K2	0.760			
K3	0.813			
K4	0.354			
A1		0.843		
A2		0.850		
A3		0.888		
S1.1			0.586	
S1.2			0.608	
S1.3			0.672	
S2.1			0.721	
S2.2			0.793	
S2.3			0.752	
S2.4			0.696	
P1				0.413
P2				0.728
P3				0.755
P4				0.611
Eigenvalues	2.225	1.728	5.613	1.234
Explained variations (%)	14.541	12.795	21.369	11.297
Naming domains	Knowledge	Attitude	Skill	Practice

#### Confirmatory factor analysis

**Figure 1:**
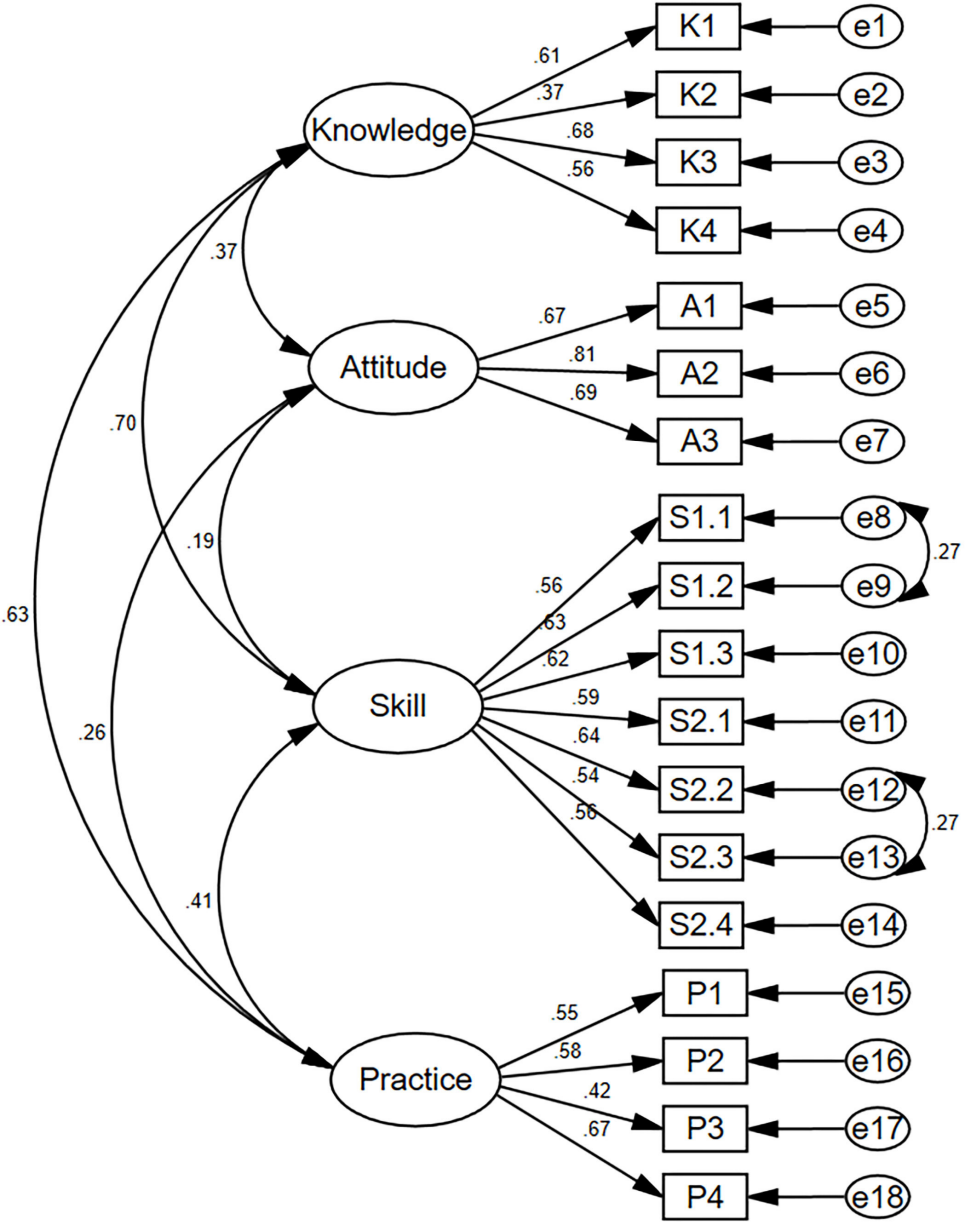
Structure equation modeling of confirmatory factor analysis for C-MLSHP-R (*n* = 229).

The structural equation modeling for C-MLSHP-R is shown in Figure 1. The fit indices demonstrated an adequate model fit (Table 4).

**Table 4:** Fit indices of confirmatory factor analysis for C-MLSHP-R (*n* = 229).

**Parameters**	**χ^2^/df**	**RMSEA**	**GFI**	**AGFI**	**CFI**	**IFI**	**TLI**	**PCFI**	**PNFI**
Measured value	1.539	0.049	0.914	0.885	0.929	0.931	0.914	0.771	0.684
Ideal value	<3	<0.05	>0.9	>0.9	>0.9	>0.9	>0.9	>0.5	>0.5

#### Convergent validity and discriminant validity

The results showed that the SFL for the majority of items was above 0.50 (except for item K2 and item P3), all CR values were above 0.60, and all AVE values were above 0.30, indicating acceptable convergent validity of C-MLSHP-R (Table 5). All the AVE square roots of domains were greater than the correlation coefficients among domains, indicating good discriminant validity of C-MLSHP-R (Table 6).

**Table 5:** Convergent validity for C-MLSHP-R (*n* = 229).

**Domains**	**Items**	**SFL**	**AVE**	**CR**
K	K1	0.608	0.319	0.643
	K2	0.372		
	K3	0.676		
	K4	0.557		
A	A1	0.666	0.527	0.768
	A2	0.809		
	A3	0.694		
S	S1.1	0.559	0.351	0.790
	S1.2	0.625		
	S1.3	0.620		
	S2.1	0.592		
	S2.2	0.645		
	S2.3	0.542		
	S2.4	0.556		
P	P1	0.548	0.315	0.642
	P2	0.584		
	P3	0.416		
	P4	0.667		

**Table 6:** Discriminant validity for C-MLSHP-R (*n* = 229).

	**Knowledge**	**Attitude**	**Skill**	**Practice**
Knowledge	0.319			
Attitude	0.261**	0.527		
Skill	0.512**	0.149**	0.351	
Practice	0.434**	0.184**	0.314**	0.315
$\sqrt{AVE}$	0.565	0.726	0.592	0.561

^**^P <0.001; AVE, the average variance extracted.

#### Criterion-related validity

A total of 178 hypertensive patients were invited to fill in the C-MLSHP and the C-MLSHP-R at the same time. Collected data were analyzed to identify criterion-related validity. The results showed that the Pearson's coefficient between the C-MLSHP and the C-MLSHP-R was 0.797 (>0.70), and the coefficient for each domain ranged from 0.609 to 0.755 (>0.60), indicating good criterion-related validity of C-MLSHP-R (Table 7).

**Table 7:** Criterion-related validity for C-MLSHP-R (*n* = 178).

**Domains**	**Coefficient**
Medication literacy	0.797**
Knowledge	0.755**
Attitude	0.614**
Skill	0.663**
Practice	0.609**

^**^P < 0.001.

## Discussion

### The development of C-MLSHP-R

The C-MLSHP with 37 items could assess medication literacy level comprehensively, but it was time-consuming for patients to read and respond to all items, making it not appropriate for wide application and quick screening. Meanwhile, the construct validity of the total scale needed to be improved. Thus, we revised the C-MLSHP in this study based on the evaluation index system of medication literacy for hypertensive patients. For C-MLSHP-R development, a three-round focus group discussion was used to formulate the preliminary revised version (26-item) in line with the 11 sub-domains of the C-MLSHP. We used the instruction of amlodipine besylate tablet, a kind of widely used antihypertensive, instead of metoprolol sustained-release tablet in the original scale (43). For C-MLSHP-R modifications, specialists were invited to appraise the correlation between each item and its belonging domain, as well as the suitability for items. After two-round expert consultation, we deleted items with poor item–scale correlation and revised the wording of some items. Then, a revised scale with 22 items was developed. Subsequently, items were checked for understandability and clarity by interviewing hypertensive patients (44). For item simplification and testing, we adopted several statistical methods to delete or revise items that were not representative of their pertaining domain, were not independent of other domains, or were not sensitive to what they should have measured (45). Finally, the C-MLSHP-R with 18 items was identified.

### The reliability evaluation of C-MLSHP-R

The internal consistency and stability of a scale reflected its reliability (46). Cronbach's α coefficients were used to assess the degree of agreement for what all items have measured. Statistically, larger sample size will test out higher Cronbach's α coefficients with the same measure. For the split-half reliability method, all items are divided equally into two parts, and the correlation coefficients will be calculated between the scores of the two parts. However, a large difference in correlation coefficients of split-half reliability will occur when the data are not randomly distributed (47). Thus, we combined Cronbach's α coefficients with split-half reliability to test the internal consistency of C-MLSHP-R. The results showed that the Cronbach's α coefficient for the total scale was >0.80 and for the attitude domain and the skill domain was >0.70; the split-half reliability for the total scale was >0.70 and for each domain was >0.60. A previous study pointed out that Cronbach's α above 0.50 was acceptable when the number of items of its domain was <5 (39). So, the Cronbach's α coefficients for the knowledge domain (four items) and the practice domain (four items) were acceptable. Thus, the internal consistency reliability of C-MLSHP-R was acceptable. The results of test–retest reliability showed that the Pearson's correlation coefficient for the total scale was >0.80 and for each domain was >0.60, indicating acceptable temporal stability of C-MLSHP-R. Overall, the C-MLSHP-R had acceptable reliability.

### The validity evaluation of C-MLSHP-R

Validity refers to the degree of agreement between what a tool has actually measured and what it intended to measure (48). The results showed that I-CVI, S-CVI/UA, and S-CVI/Ave of the C-MLSHP-R were all within the required criterion, indicating good content validity. For construct validity, EFA and CFA were advised to test and optimize the model fit between the frameworks underpinning a scale with what the scale has measured (40). Four common domains were extracted by EFA with eigenvalues >1. The cumulative variance contribution rate was above 60%, and factor loadings of all items except for item K4 (0.354) were >0.40. Ondé et al. (49) pointed out that the item with a factor loading >0.30 should be retained to reduce the risk of omitting the contents that should have been measured by a tool. As an important content for measuring patients' medication knowledge literacy, item K4 was finally retained. For CFA, model fit in this study was evaluated from three aspects: absolute fit index (χ^2^/df, RMSEA, GFI, and AGFI), value-added fit index (CFI and IFI), and parsimonious fit index (PCFI and PNFI). Most of the fit indices of this study met the required criterion, though AGFI was <0.90 (0.85), which was considered acceptable. Convergent validity was generally assessed by SFL, CR, and AVE, of which CR values and AVE values were calculated by SFL. Generally speaking, CR values above 0.70 were considered satisfied. Previous studies indicated that the CR values >0.50 were acceptable, and the AVE values <0.50 were acceptable when all CR values were >0.60 (40, 50). Though some AVE values in this study were <0.50, all CR values were above 0.60, which was considered acceptable for convergent validity with C-MLSHP-R. Fornell–Larcker criterion was used to evaluate the discriminant validity. The results showed that the square root of AVE was greater than the correlation coefficients among domains, indicating good discriminant validity. Effective criterion was a significant indicator in evaluating the criterion-related validity of the scale. The results showed that the Pearson's coefficient between the C-MLSHP and the C-MLSHP-R was >0.70, and the Pearson's coefficients for all domains were >0.60, indicating good criterion-related validity. Overall, the C-MLSHP-R had good validity.

### The comparison of C-MLSHP-R and C-MLSHP

Compared with the 37-item C-MLSHP, the 18-item C-MLSHP-R had fewer items, decreased reliability within the acceptable range, and better validity. Although Cronbach's α coefficients and split-half reliability coefficients for most domains were smaller than those of the original scale, all the reliability estimates were still acceptable. Given that the number of items in the revised scale for all domains except for the skill domain was <5, there may be a significant loss of reliability (51). Thus, the differences between the revised scale and the original scale were considered as acceptable. The cumulative variance contribution rate of C-MLSHP-R increased from 51.42 to 60.002%, and the factor loadings for items increased from 0.320–0.631 to 0.354–0.888. All the model fit indices of C-MLSHP-R showed that the present items measured fitted better with the theoretical framework underpinning the revised scale. Namely, χ^2^/df decreased from 2.629 to 1.539, RMSEA decreased from 0.066 to 0.049, GFI increased from 0.804 to 0.914, AGFI increased from 0.777 to 0.885, IFI increased from 0.746 to 0.931, PCFI increased from 0.689 to 0.771, and PNFI increased from 0.599 to 0.684. In addition, the C-MLSHP-R had acceptable convergent validity, good discriminant validity, and criterion-related validity. Thus, the C-MLSHP-R can be a good tool to evaluate the medication literacy of hypertension patients with fewer items, better readability, and less completion time from 10–15 min to 5–8 min. All in all, the applicability and generalizability of the C-MLSHP were further optimized in this study.

This study had some limitations. First, selection bias may exist in this study, as all samples were from the city of Changsha with a convenient sampling method. Second, the one-factor loading of C-MLSHP-R was <0.40, and the AVE of three domains failed to reach the required standard of 0.50, so further improvements were necessary. Finally, the application of C-MLSHP-R across cultures and different healthcare facilities was further needed.

## Conclusion

This study revised the C-MLSHP to form the C-MLSHP-R. The 18-item C-MLSHP-R had fewer items, decreased reliability within the acceptable range, and better validity compared with the 37-item C-MLSHP. It can provide a scientific, reliable, and convenient tool to assess the medication literacy level for hypertensive patients to promote hypertension management.

## Data availability statement

The original contributions presented in the study are included in the article/Supplementary material, further inquiries can be directed to the corresponding author.

## Ethics statement

This study was approved by the Institutional Review Board of the Third Xiangya Hospital, CSU (I 21072). Written informed consent for participation was not required for this study in accordance with the national legislation and the institutional requirements.

## Author contributions

NQ conceptualized this study, collected and analyzed the data, and drafted the original manuscript. YD, ZY, and SS designed this study and revised the manuscript. HL and XL collected the data. FZ acquired the funding and reviewed the writing. ZZ administrated this project and supervised the research. All authors contributed to the article and approved the submitted version.

## Funding

This study was funded by the Youth Program of the National Natural Science Foundation of China (Project Grant Number: 72104251).

## Conflict of interest

The authors declare that the research was conducted in the absence of any commercial or financial relationships that could be construed as a potential conflict of interest.

## Publisher's note

All claims expressed in this article are solely those of the authors and do not necessarily represent those of their affiliated organizations, or those of the publisher, the editors and the reviewers. Any product that may be evaluated in this article, or claim that may be made by its manufacturer, is not guaranteed or endorsed by the publisher.

## Abbreviations

C-MLSHP: the Chinese Medication Literacy Scale for Hypertensive Patients
C-MLSHP-R: the Revised Chinese Medication Literacy Scale for Hypertensive Patients
I-CVI: Item-Content Validity Index
S-CVI: Scale-Content Validity Index
MedLitRxSE: Medication Literacy Assessment in Spanish and English
ChMLM: Chinese Medication Literacy Measurement
EFA: Exploratory Factor Analysis
CFA: Confirmatory Factor Analysis
VAS: Visual Analogue Scale
SDs: Standard Deviations
KMO: the Kaiser-Meyer-Olkin
χ^2^/df: Chi-square value degrees of freedom ratio
RMSEA: Root Mean Square Error of Approximation
GFI: Goodness of Fit Index
AGFI: Adjusted Goodness of Fit Index
CFI: Comparative Fit Index
IFI: Incremental Fit Index
TLI: Tucker-Lewis Index
PCFI: Parsimonious Comparative Fit Index
PNFI: Parsimonious Normed Fit Index
AVE: the Average Variance Extracted
SFL: Standardized Factor Loading
CR: Composition Reliability.
