# Supplementary material for: Psychometric properties and validation of the revised Chinese Medication Literacy Scale for Hypertensive Patients (C-MLSHP-R)
Source: Front Cardiovasc Med. 2022 Sep 6;9:976691. doi: 10.3389/fcvm.2022.976691 (PMC9486212; doi:10.3389/fcvm.2022.976691)
Supplement: Supplementary file 1 [file Data_Sheet_1.docx]

# Revised Chinese Medication Literacy Scale for Hypertensive Patients (C-MLSHP-R)

**Ⅰ. Knowledge part** (Please select the option that you think provides the right answer to each question. Notice: multiple selections are possible)

K1. Which of the following statements about antihypertensive treatment goals do you think are correct?

① A target blood pressure treatment goal is below 140/90 mmHg in all hypertensive patients without comorbidities.

②Poor lifestyles (e.g., smoking, high-salt diet) need to be changed while taking antihypertensives.

③A target blood pressure treatment goal is below 130/80 mmHg by patients with combined coronary artery disease or diabetes if tolerated.

④A target blood pressure treatment goal is below 150/90 mmHg in people over 65 years of age.

⑤I don't know.

K2. Which of the following conditions may have a low risk of occurrence when taking antihypertensives?

①Stroke; ②Coronary heart disease; ③Fundus hemorrhage; ④Kidney disease;

⑤I don't know

K3. Which of the following factors can affect the effect of antihypertensive drugs?

①Smoking; ②Alcohol abuse; ③High salt intake; ④Anxiety or depression;

⑤I don't know

K4. Do you know the specifics of the antihypertensive drugs you are currently taking?

①Drug name; ②Drug dosage; ③Adverse drug reactions; ④Drug expiration date;

⑤I don't know

**Ⅱ. Attitude part** (Please tick the number that matches your situation)

A1. Taking antihypertensives every day disrupts my normal work or life.

①Completely agree; ②Rather agree; ③Neutral; ④Not quite agree; ⑤Completely disagree

A2. I feel that it is too much trouble to keep taking antihypertensives every day

①Completely agree; ②Rather agree; ③Neutral; ④Not quite agree; ⑤Completely disagree

A3. It's too difficult for me to remember to take antihypertensives every day

①Completely agree; ②Rather agree; ③Neutral; ④Not quite agree; ⑤Completely disagree

**Ⅲ. Skill part** (Please tick the answer you think is correct based on the following scenario)

S1. Li Si is a hypertensive patient with frequent headaches and dizziness, and his blood pressure is 170/110 mmHg at present. Here is the prescription given to Li Si by his doctor, and please read it and answer the following four questions.

**Date：**2021-06-10 **Prescribing physician:** Zhang San **Patient:** Li Si

**Amlodipine besylate tablets 5mg once a day in the morning** (Specification 5mg/tablet)

**Metoprolol extended-release tablets 23.75mg once a day in the morning** (Specification 47.5mg/tablet)

**Please monitor blood pressure after taking the drugs and go for an outpatient follow-up visit after a month.**

S1.1 How many times a day should Li Si take amlodipine benzoate tablets?

A. 1 time; B. 2 times; C. 3 times; D. 4 times; E. I don't know

S1.2 How many metoprolol extended-release tablets should Li Si take daily?

A. 1/4 tablet; B. 1/2 tablet; C. 1 tablet; D. 2 tablets; E. I don't know

S1.3 When will Li Si next go for an outpatient follow-up visit?

A. 2021-05-10; B. 2021-06-10; C. 2021-07-10; D. 2021-07-15; E. I don't know

S2. This is partial information in the instruction for **amlodipine besylate tablets**. Please answer the following four questions based on the instruction.

**[Indications]** For hypertension, chronic stable angina pectoris.

**[Precautions]** Watch out for hypotensive symptoms after taking the drug. Patients with severe coronary artery obstructive disease or impaired liver function should take it with caution according to medical advice.

**[Adverse Reactions]** A few people may experience mild to moderate headaches, edema, and palpitations.

**[Storage]** Store in a dry and sealed place away from light.

**Translated with www.DeepL.com/Translator (free version)**

S2.1 Which disease can amlodipine besylate tablets treat?

A. Hypertension; B. Kidney disease; C. I don't know

S2.2 Does this drug have any effect on his health if Li Si has alcoholic cirrhosis?

A. No effect; B. Taking with caution; C. I don't know

S2.3 Which of the following adverse reactions may occur with amlodipine benzoate tablets?

A. Dry cough; B. Edema; C. I don't know

S2.4 Please tell Li Si how to store amlodipine benzoate tablets?

A. Put in a ventilated place; B. Avoid sunlight; C. I don't know

**Ⅳ. Practice part** (Please tick the number that matches your situation)

P1. I will actively seek medication information before taking a new antihypertensive.

① Always; ② Often; ③ Sometimes; ④ Rarely; ⑤ Never

P2. I will provide feedback to the medical staff when blood pressure is not well controlled.

① Always; ② Often; ③ Sometimes; ④ Rarely; ⑤ Never

P3. I will buy and take antihypertensives as prescribed.

① Always; ② Often; ③ Sometimes; ④ Rarely; ⑤ Never

P4. I will monitor my blood pressure regularly.

① Always; ② Often; ③ Sometimes; ④ Rarely; ⑤ Never

***Thank you for your cooperation~***
