# Supplementary material for: Psychometric properties and validation of the revised Chinese Medication Literacy Scale for Hypertensive Patients (C-MLSHP-R)
Source: Front Cardiovasc Med. 2022 Sep 6;9:976691. doi: 10.3389/fcvm.2022.976691 (PMC9486212; doi:10.3389/fcvm.2022.976691)
Supplement: Supplementary file 2 [file Data_Sheet_2.docx]

Appendix1 The Comparison of Medication literacy Evaluation Index System and Scale Items

| Domains | Sub-domains | Comparison Item | |
| --- | --- | --- | --- |
|  |  | C-MLSHP-R | C-MLSHP |
| Knowledge | Knowledge for hypertension disease | K1~K2 | K1~K3 |
|  | Knowledge for hypertension treatment | K3 | K4~K5 |
|  | Knowledge for antihypertensive medication | K4 | K6~K9 |
| Attitude | Patients’ attitude and recognition to the severity of hypertension disease, as well as the necessity to be treated and controlled | A1 | A1~A2 |
|  | Patients’ attitude to taking antihypertensive medication | A2~A3 | A3~A8 |
| Skill | Patients’ ability to do numeric calculation correctly for administered dosage of antihypertensive medication, time for medication taking, as well as time for prescription refill | S1.1~S1.3 | S1~S3 |
|  | Patients’ ability to read and comprehend the prescription and medication instruction | S2.1~S2.4 | S4~S7 |
| Practice | Antihypertensive medication information-seeking and dissemination behavior | P1 | P1~P3 |
|  | Medication use decision making behavior | P2 | P4~P6 |
|  | Adherence to antihypertensive medication taking behavior | P3 | P7.1~P7.4 |
|  | Blood pressure self-monitoring and surveillance | P4 | P8~P9.2 |
